# Deformation-based Morphometry MRI Reveals Brain Structural Modifications in Living Mu Opioid Receptor Knockout Mice

**DOI:** 10.3389/fpsyt.2018.00643

**Published:** 2018-12-03

**Authors:** Md Taufiq Nasseef, Gabriel A. Devenyi, Anna E. Mechling, Laura-Adela Harsan, M. Mallar Chakravarty, Brigitte Lina Kieffer, Emmanuel Darcq

**Affiliations:** ^1^Department of Psychiatry, School of Medicine, Douglas Hospital Research Center, McGill University, Montreal, QC, Canada; ^2^Engineering Science, Computer Science and Imaging Laboratory (ICube), Integrative Multimodal Imaging in Healthcare, CNRS, University of Strasbourg, Strasbourg, France; ^3^Department of Radiology, Medical Physics, Faculty of Medicine, Medical Center University of Freiburg, University of Freiburg, Freiburg, Germany; ^4^Department of Biological and Biomedical Engineering, McGill University, Montreal, QC, Canada

**Keywords:** MRI, *in-vivo*, mice, structural change, mu opioid receptor, anatomical

## Abstract

Mu opioid receptor (MOR) activation facilitates reward processing and reduces pain, and brain networks underlying these effects are under intense investigation. Mice lacking the MOR gene (MOR KO mice) show lower drug and social reward, enhanced pain sensitivity and altered emotional responses. Our previous neuroimaging analysis using Resting-state (Rs) functional Magnetic Resonance Imaging (fMRI) showed significant alterations of functional connectivity (FC) within reward/aversion networks in these mice, in agreement with their behavioral deficits. Here we further used a structural MRI approach to determine whether volumetric alterations also occur in MOR KO mice. We acquired anatomical images using a 7-Tesla MRI scanner and measured deformation-based morphometry (DBM) for each voxel in subjects from MOR KO and control groups. Our analysis shows marked anatomical differences in mutant animals. We observed both local volumetric contraction (striatum, nucleus accumbens, bed nucleus of the stria terminalis, hippocampus, hypothalamus and periacqueducal gray) and expansion (prefrontal cortex, amygdala, habenula, and periacqueducal gray) at voxel level. Volumetric modifications occurred mainly in MOR-enriched regions and across reward/aversion centers, consistent with our prior FC findings. Specifically, several regions with volume differences corresponded to components showing highest FC changes in our previous Rs-fMRI study, suggesting a possible function-structure relationship in MOR KO-related brain differences. In conclusion, both Rs-fMRI and volumetric MRI in live MOR KO mice concur to disclose functional and structural whole-brain level mechanisms that likely drive MOR-controlled behaviors in animals, and may translate to MOR-associated endophenotypes or disease in humans.

## Introduction

The muopioid receptor (MOR) is an inhibitory G protein-coupled receptor (GPCR) belonging to the opioid receptor family (1, 2). MOR is activated by endogenous opioid peptides and by exogeneous opiates like morphine (3). MOR activation alleviates aversive states such as physical or social pain (4–7) and drives natural and drug reward processes (2, 8). Misuse and abuse of MOR agonists may cause addiction and overdose, a main cause for the rising opioid epidemics in North America (9).

Mice lacking MOR display several behavioral alterations such as increased pain perception (7) and reduced drug reward (10). Furthermore, mice with a deletion of MOR show altered sensitivity to natural rewards, as shown by their lower motivation to eat (11) and reduced maternal attachment (12). We further demonstrated that adult MOR knockout (MOR KO) animals recapitulate core and multiple comorbid behavioral symptoms of autism, including deficient social abilities, aggressiveness and stereotyped behaviors, high anxiety, impaired motor coordination and increased sensitivity to seizures, and these behavioral symptoms are associated with anatomical alterations (13). Additionally, the gene MOR deletion in mice reshapes functional connectivity in live animals (14). In the latter study, we combined blood oxygenation level–dependent (BOLD) Resting-state (Rs) functional Magnetic Resonance Imaging (fMRI) and diffusion tractography (DTI), and found pronounced modifications of whole-brain functional connectivity (FC) with only minor changes in structural connectivity (14). Strongest perturbations occurred in connectional patterns across the reward/aversion circuitry, with predominant alterations of pain/aversion-related networks (14).

**Figure 1:**
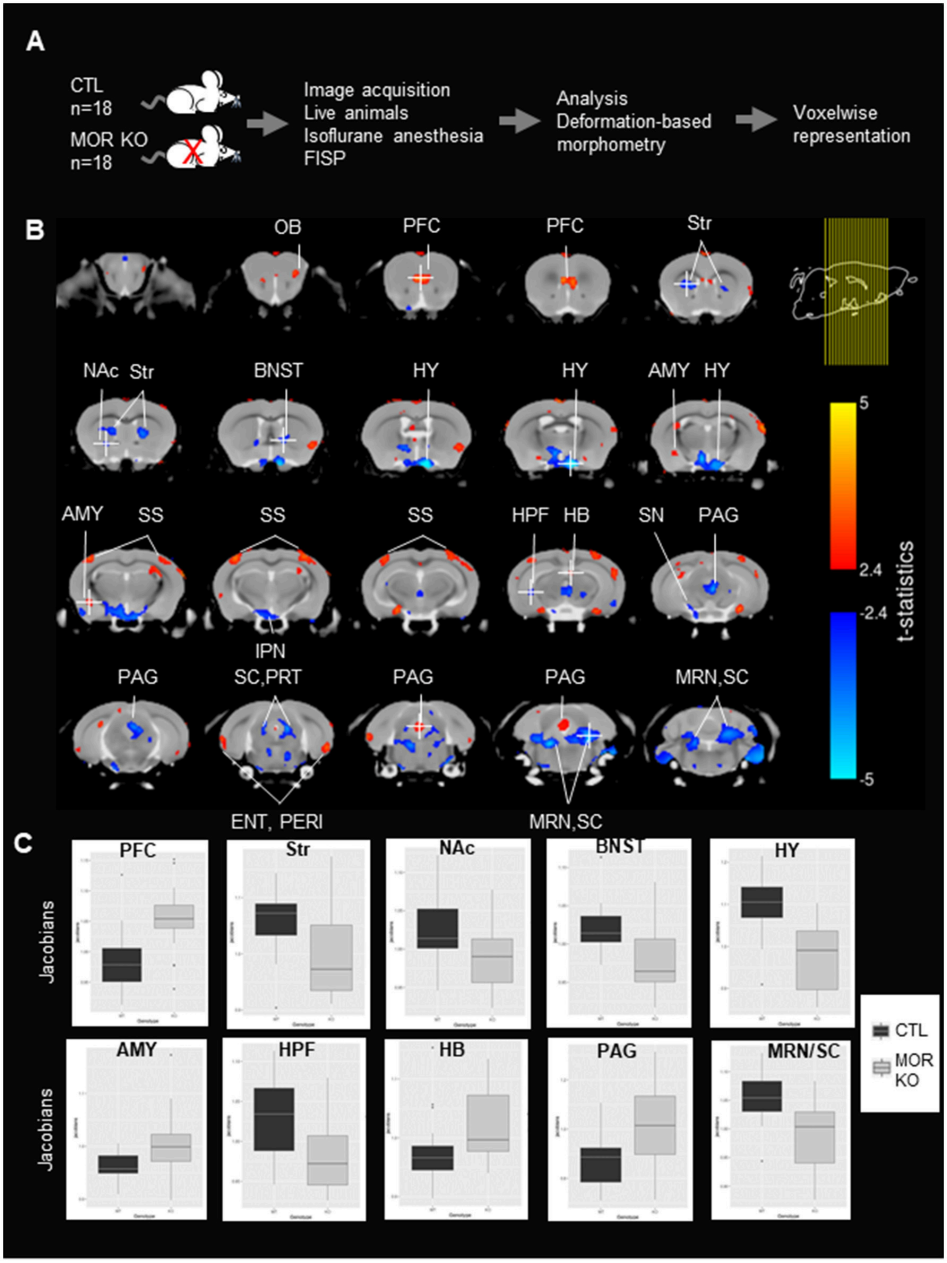
Local volumes significantly differ between controls (CTLs) and MOR Knockout (KO) mice. **(A)** Experimental timeline. Experiment was performed in CTLs and MOR KO groups (*n* = 18). MRI images were scanned following true-FISP sequences in live animals under isoflurane anesthesia. Anatomical differences in MOR KO animals were calculated using the deformation-based morphometry (DBM) and are represented voxelwise. **(B)** Significant anatomical differences (*t-statistic, p* < 0.05 and FDR correction) between CTLs and MOR KO represented on a coronal. Coronal slices are represented from anterior to posterior. Right top corner shows the location of brain mouse slices (yellow lines). Red and blue colors represent statistically significant increase and decrease of anatomical volume of MOR KO in comparison to CTLs mice. The t-statistics scale for the significant expansion are in yellow to red and for the significant contraction are in light blue to blue. The brain regions were identified using Allen brain atlas. Most regions with significant modifications are annotated. Voxels of the Str, NAc, BNST, HY, IPN, HPF, SN SC/PRT, MRN/SC are contracted, whereas voxels of the PFC, AMY, SS, HB, and PAG have expanded in mutant mice. **(C)**. Boxcar plot of relative Jacobians from both CTL and KO groups on 10 selected voxels indicated in **(B)**, and the voxel is identified with a white cross. OB, Olfactory Bulb; PFC, prefrontal cortex; NAc, Nucleus Accumbens; BNST, Bed nucleus of the stria terminalis; AMY, Amygdala; HY, hypothalamus; IPN, Interpeduncular nucleus; SS, Somatosensory cortex; HB, Habenula; TH, Thalamus; SN, Substantia Nigra; Str, striatum; HPF, hippocampal formation; SC, Superior Colliculus; PRT, Pretectal Region; PAG, Periaqueductal Gray; MRN, Midbrain Reticular Nucleus.

Because these modifications reveal circuit mechanisms underlying behavioral alterations in these mice (increased pain sensitivity and reward deficits), we sought to further explore structural abnormalities in MOR KO mice using most state-of-the-art anatomical MRI. We used high-resolution structural image acquisition in living MOR KO mice and their controls (CTLs), and identified anatomical differences caused by the deletion of MOR using a voxelwise deformation-based morphometry (DBM) analysis (Figure 1A). Our data extend a previous study (15) and reveal structural changes that parallel FC alterations that we observed in our previous study using Rs-fMRI approach in MOR KO and controls (14).

## Materials and methods

### Mice

All experiments were performed following the guidelines on animal experimentation established by the Canadian Council of Animal Care and animal protocol was approved by the Animal Care Committees of McGill University/Douglas Mental Health University Institute, Montreal, Canada (#2014-2018/7466).18 MOR KO and 18 CTLs mice were produced as described in Matthes et al. (3) and were bred at the Douglas Research Center, Montreal, Canada. Mice were kept under standard conditions at 22 ± 1°C, 60% relative humidity, and 12-h light-dark cycle with food and water available *ad libitum*. All *in-vivo* MRI experiments were performed in homozygote MOR CTLs and KO of 10–12 weeks old male mice.

### MRI anatomical acquisition

Mouse brain MRI data acquisition was performed with a 7T small animal scanner (BioSpec 70/30USR, Bruker) using 23 mm volumetric coil (Bruker). Animals were anesthetized by inhalation of isoflurane during the image acquisition. Five minutes before the start of the acquisition, mice were placed in an anesthesia chamber receiving 5% isoflurane. Then, the animals were placed into a specific animal bed system (Bruker) receiving 1.5–2% isoflurane during the whole procedure. Respiration was monitored and maintained between 45 and 70 breaths per minute using a 1025-IBP-50 Small Animal Monitoring Gating System (SA instruments). Body temperature was maintained constant at 37°C inside the magnetic bore by blowing warm air on the animal and eye lubricant was applied. MRI data were collected using 3D True-fast imaging with steady state precession (3D-true FISP) with the following parameters: matrix 128 × 128 × 64, image resolution 140 × 140 × 140 μm^3^, echo time (TE)/repetition time (TR) = 2.6 ms/800 ms. Eight radio-frequency (RF) angles (180, 0, 90, 270, 45, 225, 135, and 315 degree) were used to remove banding artifacts and the time of the whole sequence lasted for 40 min for each subject (16). Finally, root mean square (RMS) of the 8 angle acquisitions were calculated for each subject.

### Registration and analysis

Voxelwise deformation-based morphometry (DBM) was used to analyze the anatomical differences all over the brain (17, 18) following the procedure detailed in Lerch et al. (19). Briefly, mice brains were registered together through a series of linear (6 parameter followed by a 12 parameter) and nonlinear registration steps (20) to create a group-wise average. The deformation fields map the minimum deformation required at a voxel-level to map each subject to the average neuroanatomy of the group. Then, the Jacobian determinants of the deformation fields were used to measure local anatomical differences (19). These alterations could either be expansions or contractions and are dependent of the magnitude of the deformation at each voxel (19). The deformations were then mapped voxelwise by using standard tools including minc-toolkit (http://www.bic.mni.mcgill.ca/ServicesSoftware/ServicesSoftwareMincToolKit, RMINC (https://wiki.mouseimaging.ca/display/MICePub/RMINC, (rstudio (https://www.rstudio.com/, anaconda (https://repo.continuum.io/archive/ and pydpiper (https://github.com/Mouse-Imaging-Center/pydpiper. For statistical analysis, we used the general linear model, with the log of the local deformation being modeled with 18 covariates and group. Group differences were measured via the t-statistic from the linear model and corrected for multiple comparisons using FDR at 20% (RMINC, https://wiki.mouseimaging.ca/display/MICePub/RMINC RMINC was also used for single voxel boxcar plotting from Jacobians of individual subjects from each group.

## Results

### Volume changes are observed in the brain of live MOR KO mice

**Figure 2:**
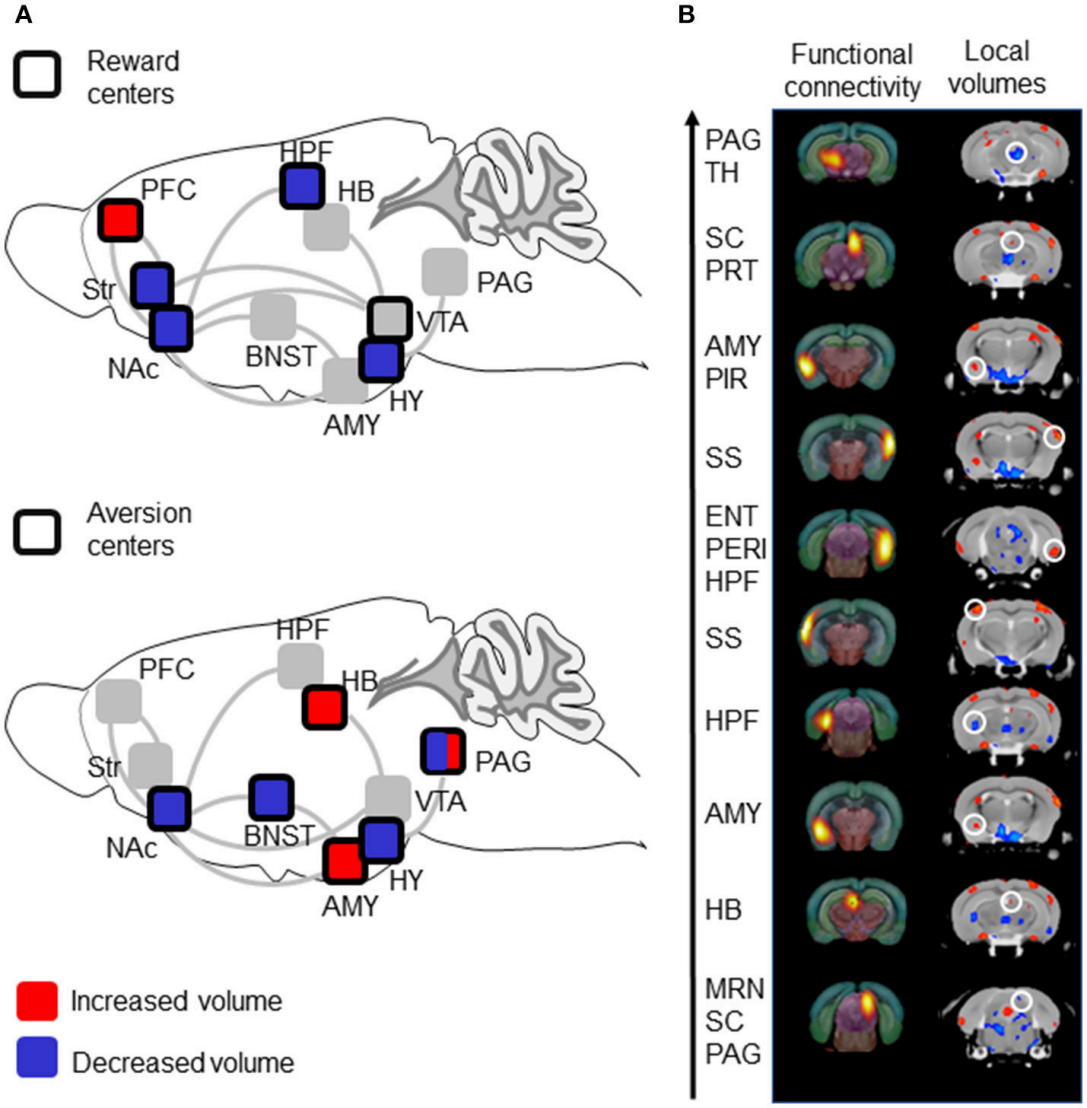
Volumetric data show decreased volumes in reward centers with contrasted effects in aversion centers, and show correspondence with previous Rs-fMRI data. **(A)** Summary of volumetric analysis. Reward centers show reduced volumes while aversion center show both reduced and increased volumes. The scheme is adapted from Darcq and Kieffer (2) and shows main brain structures involved in reward/aversion, as well as addiction. Top: Areas belonging to reward circuitry are highlighted (dark square) and volume alterations in MOR KO mice are color-coded. Bottom: Areas belonging to aversion circuitry are highlighted (dark square) and volume alterations in MOR KO mice are color-coded. PFC, Prefrontal cortex; PAG, Periaqueductal Gray; TH, Thalamus; AMY, Amygdala; SC, Superior Colliculus; PRT, Pretectal Region; PIR, Piriform area; SS, Somatosensory cortex; ENT, Entorhinal area; PERI, Perirhinal area; HPF, hippocampal; MRN, Midbrain Reticular Nucleus; HY, hypothalamus; VTA, ventral tegmental Area; BNST, Bed nucleus of the stria terminalis. **(B)** Correspondence of functional nodes (Rs-fMRI) and regions (DBM) showing significant modifications in MOR KO from our previous study (14) and in this study, respectively. The 10 nodes with highest number of statistically significant FC changes from the Rs-fMRI study are represented in the left column, and ranked (arrow) from higher (top) to lower (bottom) modifications (14). Volumetric differences between MOR KO and CTLs in this study are represented in the right column. Comparable coronal sections are displayed, and a white circle highlights local volume changes corresponding to the 10-top nodes.

The experimental flow is shown in Figure 1A. MOR KO mice and their CTLs (18 mice/group) were slightly sedated with isoflurane to avoid motion, and True-FISP sequences were used to acquire high-resolution anatomical images (16). Images were registered together through a series of linear and nonlinear fits, and deformation-based morphometry was calculated on relative Jacobians and used to compare brain local volumes (total number of voxels in the field of view = 702 720 voxels) between the two groups (see section Materials and Methods). Statistical differences between anatomical volumes from MOR KO and CTL groups were determined using parametric *t-statistic* (*p* < 0.05) and FDR correction (see section Materials and Methods), and significant anatomical differences are represented in coronal (Figure 1B) views. Local volume changes were detected in several brain areas, which we identified based on the Allen Brain Atlas. Groups of voxels showing an expansion of volume in MOR KO brains were located in the Olfactory Bulb (OB), Prefrontal Cortex (PFC), amygdala (AMY), somatosensory cortex (SS), habenula (HB) and periaqueductal gray (PAG) (Figure 1B). Sets of voxels with a local contraction were in the striatum (Str), Nucleus Accumbens (NAc), the bed nucleus of the stria terminalis (BNST), hypothalamus (HY), hippocampal formation (HPF), Interpeduncular nucleus (IPN), substantia nigra (SN), Superior Colliculus (SC) and midbrain reticular nucleus (MRN) (Figure 1B). Several groups of voxels showed a bilateral alteration, and these include the dorsal striatum, SS, MRN, HY and SC (Figure 1B). Furthermore, quantification of relative Jacobians in regional center-voxel confirmed the reduction (Str, NAc, BNST, HY, HPF, and MRN/SC) or increase (PFC, AMY, and HAB) volumes of altered regions, which were identified using DBM (Figure 1C). All these areas are known to express the receptor (21), with some of them particularly MOR-enriched (PAG, HB, AMY, Str, NAc, HY, SC), and all belong to reward/aversion centers (see summary in Figure 2A) networks (14).

## Discussion

Previous high-resolution MRI studies mainly used post-mortem brains to characterize anatomical modifications in knockout mouse lines (17, 18) or drug effects (22), to take advantage of extended scanning time without motion artifacts, and increased tissue contrast, however the brain fixation process could induce some deformation artifacts (23). We therefore conducted this study in live animals and used a reasonably long scanning time to maximize resolution while minimizing motion issues. We achieved an isotropic resolution of 140 μm with our live imaging acquisitions of 40 min, which reached about a third of what is achieved with post-mortem imaging of 702 min (isotropic resolution of 56 18). Our volumetric analysis of live MOR KO brains demonstrates significant modifications of local brain volumes in brains areas with known implication for reward and/or aversion processing.

MOR is distributed throughout the brain with enriched expression in the striatum, the medial habenula and moderate density in NAc and SN (21). These brain areas correspond to regions with reduced (Str, NAc, and SN) or increased (HB) brain volume in MOR KO mice, suggesting that MOR activity locally influences brain microstructure. Notably also the MOR deletion had significant effects in areas with poor receptor density (PFC and HPF), suggesting long distance influence of MOR activity on brain structure.

An earlier study investigated the brain of live MOR KO mice using voxel-based morphometry (15). The authors used distinct acquisition parameters that led to a resolution (125 × 125 × 300 μm) and their DBM analysis revealed significant volume increase for OB, HY, PAG, and cerebellum. Our study *(*140 × 140 × 140 μm) corroborates some of the previous findings as we also observed expanded PAG and OB local volumes, among others (Figures 1B,C). In addition, we also observed contraction of voxel volumes in several brain areas, including HPF, Str, NAc, HY, BST, SN, SC, and MRN, further supported by the observation of bilateral volume reduction for Str and HY. Our study therefore expands previous knowledge about MOR effects on brain structure. Mechanisms underlying changes in volume after the MOR deletion will require further investigations. Sasaki and colleagues showed a higher number of glial and neuronal cells in the PAG of MOR KO mice (24), which may explain some variations in brain volumes in their report and also our own study. Furthermore, it will be important in the future to determine whether volume changes arise from a developmental role of endogenous opioids, or from the lack of MOR activity at the adult age. Further imaging studies using pharmacological MOR blockade will appraise the developmental contribution of MOR activity in brain volumes.

Overall, volume changes occur throughout centers of reward and aversion processing (summarized in Figure 2A). Voxel contraction is consistently observed across reward centers, whereas both expansion and contraction are observed in aversion centers. Although this particular observation should not be overinterpreted, it is fair to propose that anatomical sites of volume changes are consistent with behavioral phenotypes of MOR KO mice. The genetic MOR deletion reduces both natural and drug rewards (2, 8), and we found reduced volumes for Str and NAc that are critical for reward processing (25). The MOR deletion also increases intracranial electrical stimulation in lateral hypothalamus (26), and bilateral reduction of the entire HY volume observed here may reflect a reduced function for this other key area of motivated behavior and reward (27). The MOR deletion increases pain perception (4, 7) and we found increased volume of PAG, critical in pain signal processing (28). Emotional behaviors are altered in MOR KO mice (29) paralleling volume changes in BNST and AMY known to regulate anxiety and stress responses (30–32).

An important aspect of the study is the observation that volumetric modifications parallel FC alterations that we previously reported using Rs-fMRI (14 and see Figure 2B). In this previous Rs-FC study, we used data-driven spatial independent component analysis (100-ICASSO) of Rs-fMRI datasets, and identified 87 functional components (clusters of voxel showing correlated and/or anticorrelated activities), which we used as nodes to establish whole brain FC matrixes for each MOR KO and CTL group and compare the two groups. A MOR-dependent FC signature emerged and, to identify most prominent alterations in MOR KO mice, we ranked these nodes based on the number of statistically significant FC changes (14). Here we considered the top-10 nodes from the Rs-fMRI study, and found that these nodes visually correspond with groups of voxels, which also show significant changes in local volumes (Figure 2B). Specifically, PAG/Thalamus (Th), SC/PRT, AMY/Piriform (PIR), right and left SS, entorhinal area (ENT), AMY, HB components from the Mechling study match regions with enhanced volumes in this study, while HPF and MRN/SC/PAG nodes match with reduced volumes (Figure 2B). Although volumetric images in this study were not co-registered with the same atlas than in Mechling et al. (14), the visual inspection therefore strongly suggest that the top-10 components with highest number of FC alterations overlap with regions showing volume modifications in MOR KO mice (Figure 2B). Concomitant structural and functional alterations for these 10 nodes, therefore, strengthen the notion that activity of these brain centers is regulated by MOR. Of note however, there is no obvious correspondence between modifications of volume size (expanded or contracted) and FC strength/diversity (enhanced or reduced), and further analysis will be necessary to determine whether variations of brain volume reflect FC plasticity upon the MOR genetic deletion.

In conclusion, progress in human anatomical MRI has greatly advanced our understanding of brain structure–function relations (33), and animal MRI is developing to allow translatable insights into drug effects (34) or vulnerability to disease (17). Future similar studies using humanized mice (35), and/or mouse exposure to chronic opiates, may pave the way to understanding mechanisms underlying the link between MOR gene variability and vulnerability to disease, or differential activities of MOR opioid agonists with distinct therapeutic profile at whole-brain level (2).

## Author contributions

MC, BK, and ED designed the study; AM, L-AH, and ED acquired the data, MN, GD, AM, L-AH, and ED performed the analysis; MN, BK, and ED wrote the manuscript. All authors contributed to manuscript revision, read and approved the submitted version.

### Conflict of interest statement

The authors declare that the research was conducted in the absence of any commercial or financial relationships that could be construed as a potential conflict of interest.
